# Risk Factors Associated with Sarcopenia Among Independently Mobile, Institutionalised Older People in the Klang Valley of Malaysia: A Cross-Sectional Study

**DOI:** 10.21315/mjms2020.27.2.13

**Published:** 2020-04-30

**Authors:** Sook Fan Yap, Nem Yun Boo, Divakara Shenoy Pramod, Zin Thaw, Siew Fun Liew, Li Fong Woo, Peak Yean Choo, Nadia Mohamad Hatta

**Affiliations:** 1 Department of Pre-Clinical Sciences, Faculty of Medicine and Health Sciences, Universiti Tunku Abdul Rahman, Selangor, Malaysia; 2 Department of Population Medicine, Faculty of Medicine and Health Sciences, Universiti Tunku Abdul Rahman, Selangor, Malaysia; 3 Department of Physiotherapy, Faculty of Medicine and Health Sciences, Universiti Tunku Abdul Rahman, Selangor, Malaysia; 4 Department of Nursing, Faculty of Medicine and Health Sciences, Universiti Tunku Abdul Rahman, Selangor, Malaysia

**Keywords:** long-term-care homes, Malaysia, older people, sarcopenia

## Abstract

**Background:**

Studies on sarcopenia among Malaysian older people (OP ≥ 60 years of age) living in the community but not in long-term care (LTC) homes have been reported previously. This study aimed to determine the prevalence of sarcopenia and its risk factors in OP in Malaysian LTC homes.

**Methods:**

This cross-sectional study was conducted with 202 independently mobile OP (males 32%) in seven LTC homes in the Klang Valley of Malaysia. Trained personnel measured their anthropometrics, body composition, gait speed, hand grip strength and timed up-and-go (TUG) duration. Criteria of the European Working Group on Sarcopenia in Older People (EWGSOP) and of the Asian Working Group for Sarcopenia were used to identify the presence of sarcopenia. The mini-nutritional assessment (MNA) was used to determine their nutritional status. Additionally, logistic regression analysis was performed to identify significant risk factors associated with pre-sarcopenia/sarcopenia.

**Results:**

Pre-sarcopenia/sarcopenia was detected in 103 (51%) OP. The significant risk factors were body mass index (BMI, weight/height^2^; adjusted odds ratio [AOR] = 0.44, *P* < 0.001), percentage of body fat (PBF; AOR = 1.26, *P* < 0.001), age group (≥ 80 years; AOR = 3.63, *P* = 0.025) and ‘at risk of malnutrition’ status (AOR = 2.63, *P* = 0.049).

**Conclusion:**

Sarcopenia is common among OP in LCT homes. The risk increases with decreasing BMI, increasing PBF, age ≥ 80 years and suboptimal nutrition status.

## Introduction

Muscle mass and strength decline rapidly with age, particularly among those ≥ 70 years of age (1–2). Decline in muscle mass coupled with suboptimal function beyond a certain level constitutes what is termed sarcopenia (3). Sarcopenia is regarded as an important geriatric syndrome that has a significant impact on physical performance, independence and the risk for falls. Multifactorial underlying causal factors in many developed countries have been reported (1–4). The reported prevalence of sarcopenia ranges from 1%–29% in community-dwelling populations and from 14%–72% in long-term care (LTC) homes in Western countries (4–9). In Asia, its prevalence was reported to be between 4.15% and 11.5% in the general communities (7, 10–11). However, data on sarcopenia among residents of Asian LTC homes are lacking.

At the time of the present study, there were only two Malaysian publications on sarcopenia in older people (OP), both on community-dwelling individuals aged ≥ 60 years (10–11). No studies had reported on sarcopenia concerning OP living in Malaysian LTC homes. The OP population in Malaysia is estimated to reach 16.3% by 2040 (12). Due to urban migration, lifestyle change and smaller families among the younger generations, many older Malaysians have opted to move into LTC homes. The present study aimed to determine the prevalence of sarcopenia and the risk factors associated with sarcopenia among OP aged ≥ 60 years in LTC homes within the Klang Valley, the most populated region in Malaysia. The ultimate objective of this study was to help healthcare providers to institute relevant preventive measures to reduce this health problem, which has a great impact on the quality of life of OP in LTC homes.

## Methods

### Study Design

This cross-sectional study was carried out over 15 months (August 2016–October 2017). Sixty-seven LTC homes identified from the internet, social workers and non-government organisation websites in the Klang Valley were approached and 28 agreed to participate. However, only seven homes were recruited as the remaining 21 homes were unsuitable, being too overcrowded and too restricted in the space needed to carry out the various measurements.

### Study Population and Measurements

The inclusion criteria were independently mobile residents, aged ≥ 60 years, who gave written informed consent. Trained medical and health personnel interviewed the 202 participants for their demographic data and measured their height and weight to derive: their body mass index (BMI, kg/m^2^); their waist circumference (WC, in cm) to determine the presence of central obesity; skeletal muscle mass and percentage of body fat (PBF) by segmental, multi-frequency bioimpedance analysis using the InBody 770 (Biospace Inc.; 13–14); gait speed (6 m walk) on a level ground (after a test run for familiarisation with the test); hand grip strength of their dominant hand using the Jamar Hand dynamometer (the best of three scores was recorded) and timed up-and-go (TUG) duration as an indicator of balance and functional mobility (15–16). Their nutritional status was also assessed using the mini-nutritional assessment (MNA) (17) and chronic illnesses were based on self-reporting and examination of prescribed medications or their doctors’ diagnoses. The presence of impaired cognition and its effects was screened using the Mini-Cog test (18) and the patient health questionnaire-2 (PHQ-2) (19), respectively.

### Determination of Sarcopenia

The criteria of the European Working Group on Sarcopenia in Older People (EWGSOP) were used (20). Individuals with a low muscle mass index, defined as appendicular skeletal mass (ASM) in kg divided by height in meter squared, of less than 2 standard deviations (SD) below the gender-specific reference of a young, healthy population (1) were categorised as having pre-sarcopenia or sarcopenia. Those who were sarcopenic were further classified into those with moderate sarcopenia (low muscle mass and low muscle strength or function) and severe sarcopenia (low muscle mass, muscle strength and function). The cut-off points for defining sarcopenia per the Asian Working Group on Sarcopenia (AWGS) were used: the appendicular skeletal mass index (ASMI, kg/m^2^) of < 7.0 kg/m^2^ in males and < 5.7 kg/m^2^ in females); handgrip strength of < 26.0 kg in males and < 18.0 kg in females and gait speed of < 0.8 m/s for both genders (21). A minimum sample size of 180 OP (with a power of 90% and a relative precision of 10%) was recruited to detect the prevalence of 60% that was reported to be present in OP living in the Malaysian community (10).

### Statistical Analysis

The SPSS statistical package (IBM, version 24.0) was used for data analysis. Descriptive analysis was carried out comparing the OP with no-sarcopenia, pre-sarcopenia and sarcopenia. In the event that very few OP had pre-sarcopenia, data of this group would be combined with the sarcopenia group for analysis compared to the no-sarcopenia group. For between group comparisons, the Chi-square test was used for categorical variables and the Student’s *t*-test or Mann-Whitney U test was used for continuous variables where appropriate. Backward logistic regression analysis was carried out to determine whether age groups, nutritional status, BMI and PBF were significantly associated with sarcopenia. All tests were two-sided. *P*-values of < 0.05 were considered statistically significant.

## Results

Of the 303 residents, 202 participated in this study; 101 (33.3%) were excluded because of moderate to severe cognitive impairment (*n* = 30), psychiatric illness (*n* = 9), severe illness (*n* = 5), impaired hearing or vision (*n* = 3), impaired mobility (wheelchair- or bed-bound; *n* = 48) or refusal to participate (*n* = 6). The mean age of the 202 subjects was 74.3 years and the majority were Chinese and female. Their mean BMI was normal while the mean ASMI, handgrip strength and gait speed were lower than normal, and the TUG duration was longer than normal (Table 1).

Table 2 shows that among the 64 males recruited, 51.6% (*n* = 33) had no sarcopenia, 4.7% (*n* = 3) had pre-sarcopenia and 43.8% (*n* = 28) had sarcopenia. Among the 138 females recruited, 47.8% (*n* = 66) had no sarcopenia, 3.6% (*n* = 5) had pre-sarcopenia and 48.6% (*n* = 67) had sarcopenia. Overall, 99 (49.0%) OP had no sarcopenia and 103 (51%) had sarcopenia (*n* = 95, 47.0%) or pre-sarcopenia (*n* = 8, 4.0%).

The mean age of the 103 pre-sarcopenia/sarcopenia OP was significantly higher compared to the no-sarcopenia group (76.8 ± 9.3 years versus 71.6 ± 8.6 years; *P* < 0.001). As shown in Table 3, a significantly higher proportion of OP were in the ‘≥ 80 years’ age group and in the ‘at risk of malnutrition’ category. They had significantly lower mean body weight, height, BMI and WC as well as lower frequency of diabetes mellitus. There was no significant difference in the gender distribution, mean PBF or other comorbidities between the two groups. Backward logistic regression analysis showed that the only significant risk factors associated with pre-sarcopenia/sarcopenia were BMI, PBF, the ≥ 80 years age group and ‘at risk of malnutrition’ status (Table 4). The percentage of cases predicted correctly by the regression model was 85.6% and the Hosmer-Lemeshow goodness of fit test had a *P-*value of *P* < 0.001. The area under the receiver operating curve (ROC) for the multiple logistic regression model was 0.925 (95% CI: 0.888, 0.963). TUG duration could only be assessed in 147 (72.8%) OP (no-sarcopenia, *n* = 67; pre-sarcopenia/sarcopenia, *n* = 80). The no-sarcopenia OP had significantly shorter median TUG duration (15.7 s; range: 7.0–78.0) than the pre-sarcopenia/sarcopenia OP (19.1 s; range: 5.0–109.0; *P* = 0.021).

## Discussion

This was the first Malaysian study on the prevalence and risk factors associated with sarcopenia among OP in a number of LTC-homes and the first to report on TUG duration in this group. Due to the over-crowdedness and limited space in many of the homes, we were unable to recruit more centres or more patients. This lack of space in the homes also means that the residents did not have much space for physical activities, the lack of which could contribute to the development of sarcopenia.

We did not include bodyweight, WC measurements or diabetes mellitus as potential confounders in the logistic regression analysis. This was because BMI was calculated from bodyweight and a large number of participants did not have WC measurements (*n* = 32, 15.8%) or information on their diabetes mellitus status (*n* = 36, 17.8%). The results of the present study showed that OP of 80 years of age and above living in these LTC homes had 3.63 times the risk of sarcopenia than non-sarcopenia OP. Furthermore, those in the category of ‘at risk of malnutrition’ had 2.63 times the risk and every 1% increase in PBF had a 1.26 times increased risk while every one unit decrease in BMI had a 56% increased risk of sarcopenia. The large area under the ROC for decreasing BMI values (0.804) indicates that it had high sensitivity for predicting sarcopenia in these OP. The most common cause of decreasing BMI is sub-optimal nutrition and the major implication of our findings is that improvement of the nutritional status could be an important strategy for reducing the risk of sarcopenia in OP living in LTC homes.

In this study, the prevalence of sarcopenia among LTC OP was 47%, which is much higher than the 14%–33% reported by the International Sarcopenia Initiative (EWGSOP and IWGS) (4). In two studies on community-dwelling OP in Malaysia in 2013 (10) and 2017 (11), the reported prevalence was 59.8% and 50.5%, respectively, which were fairly similar to our results. However, we suspect that we probably underestimated the actual figure, as about 48% of the LTC residents who were excluded had impaired mobility.

Similar to the reports from other countries, our study confirmed that the ≥ 80 years age group (1, 20–23) and lower BMI (24–25) were significant risk factors. Although we found ‘at risk of malnutrition’ was a significant risk factor, malnutrition itself was not a risk factor in our cohort. One possible explanation could be that our study was under-powered to detect malnutrition. We were also unable to use logistic regression analysis to determine whether diabetes mellitus and other comorbidities were risk factors associated with sarcopenia, as reported elsewhere (26–27), because of the incomplete information stated above. Unlike previous reports (8–9), gender was not a significant risk factor in our cohort. We were also unable to determine whether the ethnic group was a risk factor because the majority of our subjects were Chinese.

TUG duration assesses function and mobility, providing an objective measure of risk for falls. Any individual with TUG duration > 13.5 s has a high risk for falls (28). Given that the median duration of the TUG of both the no-sarcopenia and sarcopenia subjects in this study exceeded this cut-off level, thus, there is a need to institute preventive physiotherapy for all LTC OP to minimise falls. Furthermore, among no-sarcopenia subjects, a proportion actually had decreased grip strength and/or gait speed, suggesting that loss of function precedes loss of muscle mass, an observation that has been reported by other investigators (29–30).

The main limitations of this study include the small sample size, inability to measure WC in 15.8% of the subjects and omission of a hip circumference (HC) measurement to determine its association with sarcopenia (29–30). HC reflects the sizes of the skeletal frame, adipose tissue and muscle mass in the gluteal region, providing a better assessment of the nutritional status of a person than WC alone. However, logistically, it is more difficult to measure accurately in the frail and thin OP than WC.

## Conclusion

Sarcopenia is common among independently mobile OP in Malaysian LTC homes. Age ≥ 80 years, sub-optimal nutritional status, increasing PBF and decreasing BMI were significant risk factors. Improving their nutrition status may be an important strategy for preventing or minimising the severity of this problem.

## Figures and Tables

**Table 1:** Demographics, anthropometrics, body composition and physical functions of study subjects

Variables	Total

	*N* = 202
Age (Year)	
Mean (± SD)	74.3 (9.3)
range	60–99
Age groups (%)	
60–69 years	77 (38)
70–79 years	58 (29)
≥ 80 years	67 (33)
Females (%)	138 (68)
Ethnic groups (%)	
Chinese	188 (93)
Indian	9 (5)
Malay	4 (2)
Others	1 (0)
Physical parameters, mean (± SD)	
Body weight, kg	54.5 (13.7)
Body height, m	1.5 (0.1)
BMI, kg/m^2^	23.0 (5.0)
Range	12.4–40.1
Body composition, mean (± SD)	
ASM, kg	14.6 (5.0)
ASMI, kg/m^2^	6.0 (1.5)
% of body fat	*N* = 201
	30.8 (11.0)
Physical function, mean (± SD)	
Handgrip strength, kg	16.0 (7.1)
Gait speed, m/s	0.7 (0.3)
Timed up-and-go, s	*N* = 147
Median (IQR)	17.9 (12.3, 25.0)

Notes: BMI = body mass index; ASM = appendicular skeletal muscle; ASMI = appendicular skeletal mass index (ASM/ht^2^); SD = standard deviation; IQR = interquartile range

**Table 2:** The comparison of BMI, muscle strength, and functions of males and females with and without sarcopenia based on criteria of EWGSOP and AWGS

Variables	No sarcopenia	Pre-sarcopenia	Sarcopenia	

			Moderate	Severe
**Males**				
	
	*N* = 33	*N* = 3	*N* = 10	*N* = 18
	
BMI, kg/m^2^ mean (± SD)	26.3 (4.1)	20.8 (2.1)	21.5 (3.4)	19.5 (3.3)
ASM, kg median (range)	21.4 (16.7–29.2)	13.3 (12.0, 19.4)	15.2 (9.9–17.1)	16.1 (9.4–20.9)
ASMI, kg/m^2^ median (range)	7.9 (7.0–9.8)	5.4 (5.2–6.9)	6.1 (5.1–6.6)	6.1 (4.3–7.0)
Handgrip strength, kg median (range)	27.0 (10.0–34.0)	27.0 (27.0–29.0)	20.8 (15.0–24.0)	19.0 (11.0–24.0)
Gait speed, m/s median (range)	0.8 (0.3–1.4)	0.9 (0.8–1.3)	0.8 (0.8–1.0)	0.5 (0.3–0.7)
**Females**				
	
	*N* = 66	*N* = 5	*N* = 13	*N* = 54
	
BMI, kg/m^2^	25.3 (5.3)	24.3 (2.4)	20.1 (3.5)	20.6 (3.9)
ASM, kg median (range)	14.7 (11.4–31.7)	12.4 (11.5–13.3)	9.5 (4.9–12.8)	10.4 (5.2–14.3)
ASMI, kg/m^2^ median (range)	6.1 (5.7, 13.2)	5.4 (5.4–5.7)	4.7 (2.3–5.5)	4.8 (2.4–5.7)
Handgrip strength, kg median (range)	14.3 (5.0–29.0)	19.0 (18.0–24.0)	13.0 (4.0–20.0)	10.3 (2.0–17.0)
Gait speed, m/s median (range)	0.7 (0.3–1.4)	1.2 (0.8–1.4)	0.9 (0.6–1.4)	0.5 (0.2–0.7)

**Table 3:** A comparison of demographics, anthropometrics, nutritional status and comorbidities between subjects with and without pre-sarcopenia/sarcopenia

Variables	No sarcopenia *N* = 99	Pre-sarcopenia or sarcopenia *N* = 103	*P*-values
Age groups (%)			
60–69	49 (50)	28 (27)	< 0.001*
70–79	32 (32)	26 (25)	
≥ 80	18 (18)	49 (48)	
Gender (%)			
Male	33 (33)	31 (30)	
Female	66 (67)	72 (70)	0.621*
Nutritional status (%)	*N* = 82	*N* = 91	
Normal	51 (62)	22 (24)	< 0.001*
At risk of malnutrition	24 (29)	58 (64)	
Malnourished	7 (9)	11 (12)	
Anthropometrics, mean (± SD)	*N* = 99	*N* = 103	
Weight, kg	63.0 (12.6)	46.4 (9.0)	< 0.001#
Height, m	1.6 (0.1)	1.5 (0.1)	< 0.001#
BMI, kg/m^2^	25.6 (4.9)	20.4 (3.6)	< 0.001#
		*N* = 102	
PBF, %	31.7 (11.7)	29.9 (10.2)	0.080#
WC, cm	*N* = 81	*N* = 89	< 0.001#
	94.1 (11.7)	83.6 (12.8)	
Comorbidities (%)			
Cognitive impairment	*N* = 77	*N* = 74	0.226*
	22 (29)	28 (38)	
Affective impairment	*N* = 79	*N* = 83	0.740*
	21 (27)	24 (29)	
Joint pain	*N* = 82	*N* = 85	0.879*
	25 (31)	25 (29)	
Diabetes mellitus	*N* = 82	*N* = 84	0.021*
	31 (38)	18 (21)	
Stroke	*N* = 83	*N* = 85	0.739*
	7 (8)	6 (7)	

Notes: SD = standard deviation; BMI = body mass index; WC = waist circumference; PBF = percentage of body fat;

* analysis by Chi-square;

# analysis by Student’s *t*-test

**Table 4:** Logistic regression analysis of potential risk factors associated with pre-sarcopenia/sarcopenia

Variables	Crude OR (95% CI)	*P*-value	AOR (95% CI)	*P*-value
Age groups				
60–69 years	Reference		Reference	
70–79 years	1.42 (0.71, 2.95)	0.321	1.76 (0.60, 5.17)	0.302
≥ 80 years	4.76 (2.33, 9.71)	< 0.001	3.63 (1.18, 11.20)	0.025
Nutritional status				
Normal	Reference		Reference	
At risk of malnutrition	5.60 (2.81, 11.17)	< 0.001	2.63 (1.01, 6.90)	0.049
Malnourished	3.64 (1.25, 10.64)	0.018	0.64 (0.13, 2.99)	0.570
BMI (kg/m^2^)	0.74 (0.68, 0.81)	< 0.001	0.44 (0.33, 0.58)	< 0.001
PBF (%)	0.99 (0.96, 1.01)	0.249	1.26 (1.15,1.38)	< 0.001

Notes: BMI = body mass index; PBF = percentage of body fat; OR = odds ratio; CI = confidence intervals
